# Non-pharmacological Interventions for Anxiety and Depression in Adults With Inflammatory Bowel Disease: A Systematic Review and Meta-Analysis

**DOI:** 10.3389/fpsyg.2020.538741

**Published:** 2020-11-05

**Authors:** Suja P. Davis, Linda P. Bolin, Patricia B. Crane, Jamie Crandell

**Affiliations:** ^1^School of Nursing, University of North Carolina at Charlotte, Charlotte, NC, United States; ^2^College of Nursing, East Carolina University, Greenville, SC, United States; ^3^Department of Biostatistics, Gillings School of Global Public Health, University of North Carolina at Chapel Hill, Chapel Hill, NC, United States

**Keywords:** inflammatory bowel disease, non-pharmacological interventions, anxiety, depression, meta-analysis

## Abstract

**Objectives:** To assess the published randomized controlled trials (RCT) of non-pharmacological interventions systematically and to synthesize the evidence of these interventions for the management of anxiety and depression in adults with inflammatory bowel disease (IBD).

**Background:** Anxiety and depression are common symptoms in adults with IBD and can have many negative outcomes on their quality of life (QOL). Non-pharmacological interventions for anxiety and depression are important to improve the adaptive strategies of adults with IBD. Previously published reviews of non-pharmacological interventions to mitigate anxiety and depression in those with IBD have resulted in inconclusive evidence. This review is aimed to fill that gap.

**Design:** Systematic review and meta-analysis.

**Method:** Using a PRISMA diagram, English-language RCT published were searched using combined keywords of inflammatory bowel disease, Crohn's disease, ulcerative colitis, randomized controlled trial, anxiety, and depression. The Cochrane risk of bias tool is utilized to assess the methodological quality of each study. A meta-analysis of RCTs was conducted using Comprehensive Meta-Analysis (CMA) software.

**Results:** The final review included 10 studies. The overall risk of bias of the selected studies varied from low risk in three studies, some concerns in four of the studies, and high risk of bias in three of the studies. Interventions included cognitive-behavioral therapy, mindfulness-based therapy, breath–body- mind –workshop, guided imagery with relaxation, solution-focused therapy, yoga, and multicomponent interventions. The pooled evidence from all non-pharmacological interventions showed that these interventions significantly helped to reduce anxiety, depression, and disease specific quality of life (QOL) in adults with IBD compared to control groups. However, the effect sizes are small. The pooled standardized mean difference (SMD) was −0.28 (95% CI [−0.47, −0.09], *p* = 0.004) for anxiety, −0.22 (95% CI [−0.41, −0.03], p = 0.025) for depression and 0.20 (95% CI [0.004, 0.39], *p* = 0.046) for disease specific QOL.

**Conclusion:** The addressed non-pharmacological interventions were multifaceted and demonstrated positive effects on anxiety and depression, and QOL in those with IBD. Healthcare providers can facilitate a discussion with adults with IBD about the availability of these interventions to mitigate their anxiety and depression and to improve their QOL.

## Introduction

Inflammatory bowel disease (IBD) is an immune-mediated chronic gastrointestinal illness that encomapses Crohn's disease (CD) and ulcerative colitis (UC). IBD is characterized by remission (disease-free interval) and relapse (active disease) (van de star and Banan, 2015). About 2.2 million Europeans, 1.5 million Americans, and more than 100,000 others worldwide are affected by IBD (Ananthakrishnan, 2015). IBD prevalence rate is rising in Asia, Africa, and South America, where formerly IBD was considered a rare disease (Ananthakrishnan, 2015). Clinical manifestations of IBD include diarrhea, fever, fatigue, pain in the abdomen, reduced appetite, loss of weight, and blood in stools. While most note the physical symptoms, psychological symptoms experienced by those with IBD and the associated treatments are prevalent with 35% reporting anxiety symptoms and 22% depressive symptoms (Nahon et al., 2012; Panara et al., 2014; Neuendorf et al., 2016).

Multiple factors can contribute to the development of anxiety and depression in adults with IBD. Active disease is the main factor, followed by female gender and socioeconomic deprivation or low socioeconomic status (Nahon et al., 2012; Clark et al., 2014; Panara et al., 2014). Other contributing factors depression and anxiety in patients with IBD are the adverse effects and associated mood disturbances of treatments such as corticosteroids, biologics and immunomodulators, and the fear of side effects (Nahon et al., 2012; Mikocka-Walus et al., 2016; Choi et al., 2019). Addressing these psychological symptoms is important because psychological stress is known to escalate disease activity in adults with IBD (Sajadinejad et al., 2012).

Psychological stress mediates IBD through the brain-gut axis. Stress leads to the secretion of peripheral corticotrophin-releasing factor, which contributes to the gut becoming more prone to inflammation by (a) altering intestinal motility and (b) increasing intestinal permeability, resulting in a reduction of the mucosal barrier action (Sajadinejad et al., 2012). Stress also leads to the dysfunction of the intestinal immune system in two ways: (1) the intestinal mucosa is attacked, and (2) the hypothalamic-pituitary-adrenal axis is activated. This results in the secretion of stress hormones (e.g., cortisol) and the release of inflammatory mediators (e.g., cytokines) (Sajadinejad et al., 2012). Caneo et al. (2016) reported an increased risk for depression in adults with physical ailments involving systemic inflammation compared to those without inflammation. Consequently, the risk for depression is high in adults with IBD due to systemic inflammation. Thus, the psychological symptoms of IBD, such as anxiety and depression, is likely related to a multitude of factors that include treatments, adverse effects of the pharmacological interventions, stress, and inflammation (Bannaga and Selinger, 2015; Keefer and Kane, 2017).

Anxiety and depression are inversely related to health behaviors, and quality of life (QOL) in adults with IBD (Faust et al., 2012). Several investigators have noted that anxiety and depression explain medication non-adherence among individuals with IBD (Nahon et al., 2012; Spekhorst et al., 2016). This is concerning, as medication non-adherence may lead to IBD exacerbations resulting in an increase of distressful symptoms. More importantly, results from a longitudinal study of more than 2,000 adults with IBD revealed a significant positive association between disease recurrence, and symptoms of depression or anxiety (Mikocka-Walus et al., 2016). Care for those with IBD should include not only treating physical symptoms but also the psychological manifestations of anxiety and depression.

Interventions to address depression and anxiety are important to improve the adaptive strategies of adults with IBD (McCombie et al., 2015) and should include both pharmacological and complementary interventions. A previous systematic review by Fiest et al. (2016) evaluated the outcomes of pharmacological and psychological interventions on depression and anxiety in adults with IBD, and their results revealed only one study which addressed pharmacological interventions to address anxiety. This systematic review did not support psychological interventions to manage anxiety and depression. However, the authors (Fiest et al., 2016) acknowledged the high risk of bias with this study. Previously published reviews of non-pharmacological interventions to mitigate anxiety and depression in adults with IBD resulted in inconclusive evidence. Most of these studies were more than 10 years old, and none focused on meta-analysis of non-pharmacological interventions to mitigate anxiety or depression among those with IBD. Hence, there is a need for synthesizing new evidence on the effectiveness of non-pharmacological interventions to mitigate anxiety and depression in those with IBD.

## Research Aims

This review aimed to focus on: (1) determine the types of non–pharmacological interventions published in literature to manage anxiety and depression among adults with IBD, (2) analyze the effectiveness of these non–pharmacological interventions to manage anxiety, depression and QOL in adults with IBD. We formulated the following research question: Are non-pharmacological interventions more effective compared to control groups in managing anxiety and depression, and improving QOL in adults with IBD?

## Methods

### Search Strategy

**Figure 1:**
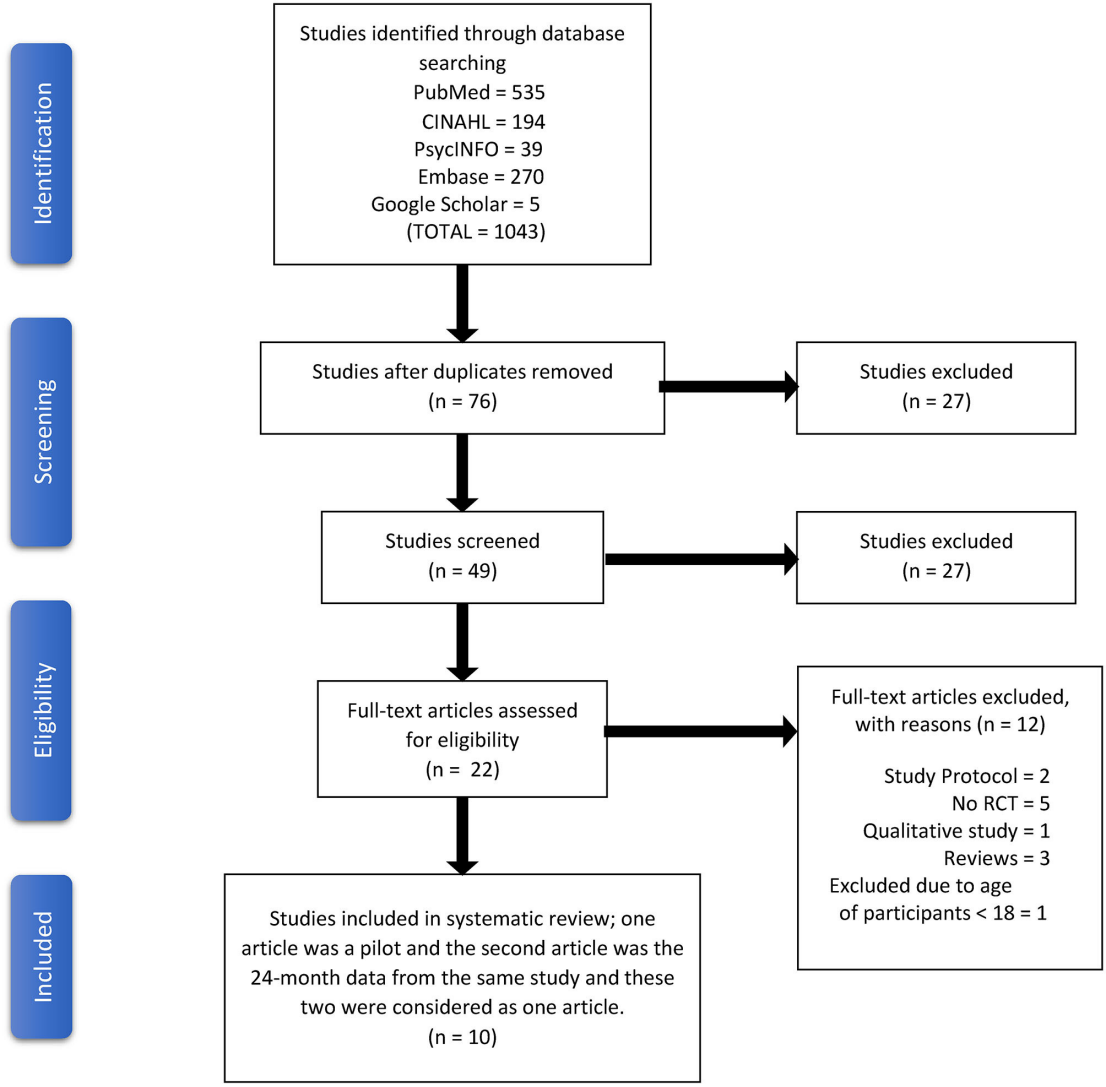
PRISMA diagram.

The Preferred Reporting Items for Systematic Reviews and Meta-Analyses (PRISMA; Moher et al., 2009) was used as a framework to guide the search strategy. Using the databases of PubMed, CINAHL, EMBASE, PsychInfo, and Google Scholar, a search for English-language articles on randomized clinical trials (RCT) and IBD published with the primary or secondary outcome of depression and/or anxiety was conducted till December, 2019. A Boolean search strategy (Schuler et al., 2008) using combined keywords of *inflammatory bowel disease, Crohn's disease, ulcerative colitis, randomized controlled trial, anxiety*, and *depression* yielded 1,043 studies. Each database was searched to identify research articles by customizing the same medical subject heading (MeSH) terms and key words in consultation with a research librarian. The search strategy details for each of the database are included in Supplementary Table 1. An additional search was conducted manually by using the citation lists of the retrieved studies to identify other relevant articles related to the Research Topic. Studies that matched the criteria for inclusion were retained for this systematic review. The PRISMA flow diagram (Figure 1) summarizes the processes of the systematic review.

### Inclusion and Exclusion Criteria

This review had the following inclusion criteria: (1) RCTs in English-language in published journals that focused non-pharmacological components of interventions to mitigate anxiety, depression and QOL among adults with IBD, (2) measured anxiety, depression and QOL as an outcome at both before and after intervention, (3) studies that include adults who are 18 years or above with a diagnosis of ulcerative colitis or Crohn's disease, and (4) participants with IBD in remission or relapse were included for the study. This review excluded the studies of : (1) pediatric and adolescent population (2) expert opinion and general reviews, and (3) RCTs in other languages.

### Quality Appraisal

**Figure 2:**
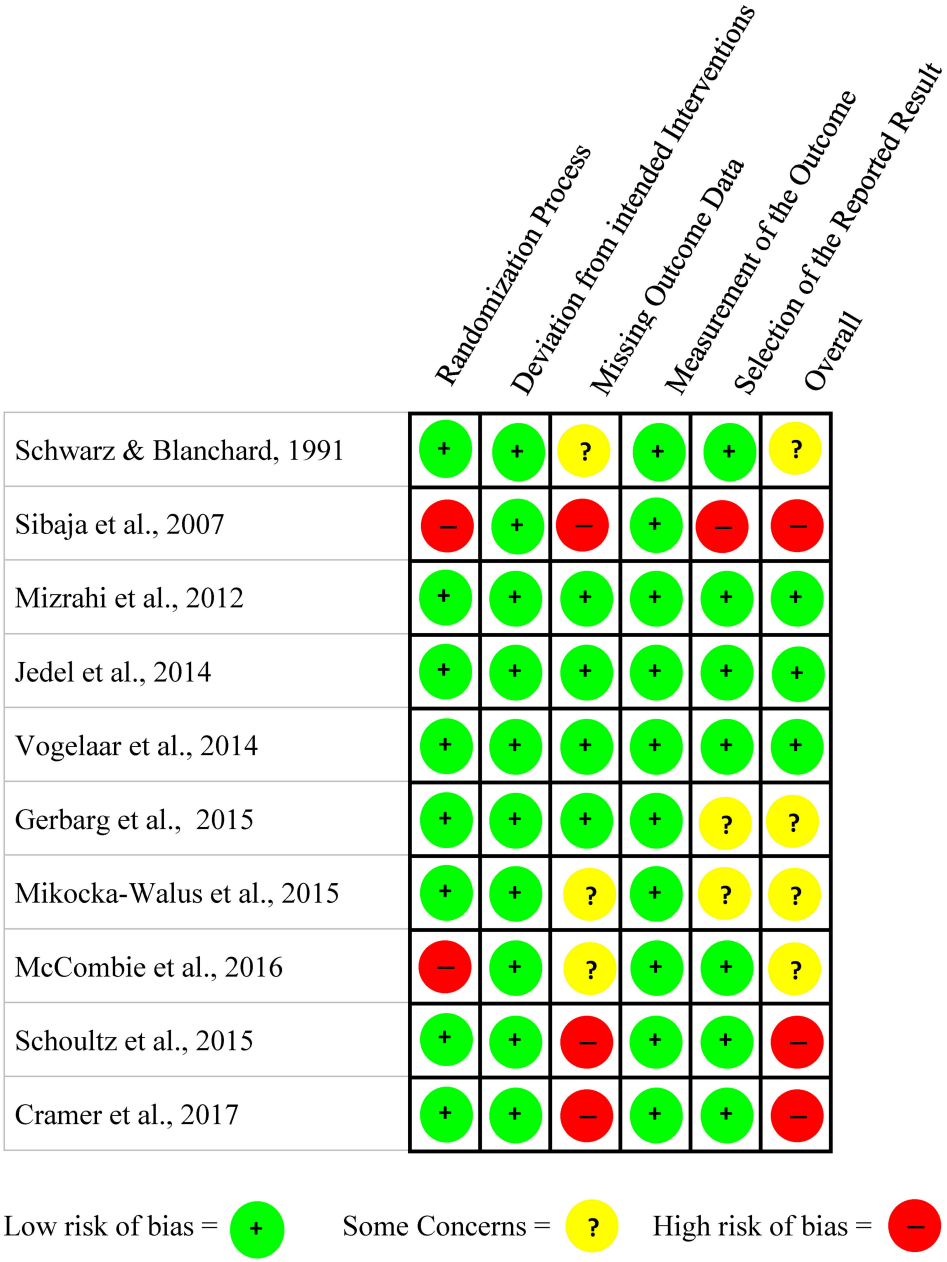
Methodological quality evaluation of RCTs using Cochrane risk assessment tool.

Independent assessment of the methodological quality of each RCT was conducted by the first and second authors using the Cochrane risk of bias tool, version 2 (RoB 2; Sterne et al., 2019). The RoB 2 is designed for quality appraisal of RCTs based on five domains with pre-determined questions to generate an algorithm for each of the domains to indicate the bias for each study (Sterne et al., 2019). For each study, the results of the risk of bias were categorized as low risk (color coded as green), some concerns (color coded as yellow) and high risk (color coded as red) for each domain as well as for the overall risk. If the risk assessment of all the domains resulted in low, then the study's overall risk is considered to be low (Sterne et al., 2019). The selected studies' overall risk of bias varied from a low risk in three studies (Mizrahi et al., 2012; Jedel et al., 2014; Vogelaar et al., 2014), some concerns in four of the studies (Schwarz and Blanchard, 1991; Gerbarg et al., 2015; Mikocka-Walus et al., 2015; McCombie et al., 2016), and three studies showed a high risk of bias (Sibaja et al., 2007; Schoultz et al., 2015; Cramer et al., 2017). A consensus was noted among the authors while evaluating the quality appraisal of the nine selected studies. The quality appraisal results of the studies are reported in Figure 2.

### Data Extraction

The first and the second author independently extracted the data, which was verified by the third author after reading all the articles that matched the criteria for inclusion. The first and second authors examined the extracted data and came to an agreement after discussion. The authors organized the extracted data in the following: (1) author name, study design; (2) details of sample (sample size in intervention and control groups, type of IBD); (3) details of interventions; (4) duration of intervention and follow up (5) instruments used; and (6) major results of outcomes.

### Analyses

The search yielded 1,043 studies; 76 articles were retained after the duplicates were removed. We excluded 27 studies after screening their abstracts. From the remaining 49 articles, we removed an additional 27 studies following the exclusion and inclusion criteria. The remaining 22 were assessed for eligibility. Eleven articles were eligible for the final synthesis. Out of these 11, one article was a pilot (Mikocka-Walus et al., 2015) and the second article was a 24-months data set analysis from this same pilot study (Mikocka-Walus et al., 2017). The end resulted in 10 unique studies. Because the intervention was the same, these two articles were considered one study and the parent study article authored by Mikocka-Walus et al. (2015) was selected for the final review. These steps are summarized in a PRISMA diagram (Figure 1).

Each article was analyzed by the sample characteristics, study interventions, length of time for interventions, along with primary and secondary outcome measures related to anxiety and depression (see Table 1). Quantitative data were sufficient enough to conduct meta-analysis for seven (Mizrahi et al., 2012; Jedel et al., 2014; Gerbarg et al., 2015; Mikocka-Walus et al., 2015; Schoultz et al., 2015; McCombie et al., 2016; Cramer et al., 2017) of the 10 studies to evaluate the efficacy of non-pharmacological interventions to mitigate anxiety and depression in adults with IBD. Three studies had (Schwarz and Blanchard, 1991; Sibaja et al., 2007; Vogelaar et al., 2014) limited data on anxiety and depression to include in our meta-analysis. The primary author attempted to contact the corresponding authors of two studies (Sibaja et al., 2007; Vogelaar et al., 2014) without any success. The contact information for the corresponding author was not included in this third study (Schwarz and Blanchard, 1991). Therefore, all three studies were synthesized narratively.

**Table 1:** Summary of study findings.

**References**	**Study method/Country**	**Type of sample**	**Study sample (1) Total sample (total enrolled/completed) (2) Intervention/ Control**	**Intervention component and type**	Duration of intervention period and *Follow up	**Instruments**	**Outcome results**
Schwarz and Blanchard (1991)	RCT USA	All IBD patients (UC and Crohns)	(1) 21/21 (2) 11/10	**Multicomponent Intervention Procedure** *Muscle relaxation *Biofeedback *Training on cognitive coping skills *IBD education	One hour face to face sessions twice a week for 4 weeks, followed by weekly sessions for 4 weeks	**The State Trait Anxiety Inventory (STAI)-**measured anxiety **Beck depression inventory**—measured depression	Although anxiety and depression scores lowered after the intervention, the change was not statistically significant.
Sibaja et al. (2007)	RCT Spain	All IBD patients (UC and Crohns)	**Initial** (1) 57/57 (2) 33/24 **At 3 months** (2) 33/0 **At 6 months** (2) 22/0 **At 12 months** (2) 18/0	**CBT (face to face group therapy)** *Information about IBD *Training on coping skills *Training on problem solving *Relaxation techniques Social skill improvement *Methods of distraction * Strategies to address cognitive restructuring techniques	2 h face to face group session for 10 weeks *A document with important content and tasks were given after each session * A relaxation tape or CD was provided	**Beck depression inventory**—measured depression **Hospital anxiety and depression scale (HADS)**—measured anxiety and depression	(+) outcomes with anxiety (*p* <0.001) and depression (*p* <0.001) till 12 months among CBT group
Mikocka-Walus et al. (2015)	RCT Australia	IBD patients in remission	**Initial** 176/174 (2) 90/84 **At 6 months** (2) 51/65	**CBT (both face to face and computerized)** The CBT program included *Education about IBD and CBT *Relaxation and stress reduction *Interpretation of unpleasant events *Restructuring the events that led to negative thoughts Techniques to overcome avoidance *Strategies to improve coping skills *Empowering themselves *strategies to improve relationships *Techniques to improve distraction	The CBT was offered 2 h each week for 10 weeks. Both face to face group therapy and computer based CBT were available. *No follow up noted	**HADS**—measured anxiety and depression **The State Trait Anxiety Inventory (STAI)-** measured anxiety **The short form 36 health status questionnaire (SF-36)—**measured QOL **Disease activity**—CDAI measured Crohn's disease and SCCAI measured disease activity of ulcerative colitis. **Diseases activity** is also measured by CRP, hemoglobin, platelet and white cell count.	*(+) outcomes with trait anxiety (*p* = 0.042) and depression (*p* = 0.018) till 12 months among CBT group.
Mikocka-Walus et al. (2017)	RCT Australia	IBD patients in remission	(1) 176/174 (2) 30/45	**CBT (both face to face and computerized)** CBT techniques were same as that of the previous study (Mikocka-Walus et al., 2015)	Same as that of the previous study (Mikocka-Walus et al., 2015) *No follow up noted	**HADS**—measured anxiety and depression **STAI-** measured anxiety **SF-36—**measured QOL **Disease activity**—CDAI measured Crohn's disease and SCCAI measured disease activity of ulcerative colitis. **Diseases activity** is also measured by CRP, hemoglobin, platelet and white cell count.	*Both state and trait anxiety trended down among CBT group at 24 months, but not statistically significant. Depression scores did not change at 24 months. * No change in anxiety and depression among ‘in need” patients at 24 months.
McCombie et al. (2016)	RCT Newzealand	All IBD patients	**Initial** (1) 131/100 (2) 113/86 **At 3 months** (2) 65/78 **At 6 months** (2) 53/66	**Computerized CBT** The CCBT sessions included *Relaxation techniques Influence of the thoughts on behavior, *Coping skills *Communication skills *Distraction techniques	A total of 8 sessions with 62 resources in the CCBT *Email reminder once per week for 8 weeks and text messaged at 6 weeks	**HADS**—measured anxiety and depression **IBDQ—**measured IBD specific HRQOL **SF-12** - measured general HRQOL	Anxiety and depression were not associated with CCBT.
Schoultz et al. (2015)	Pilot RCT UK	IBD patients	**Initial** 21/22 **Post intervention** 12/12 **At 6 months** (2) 12/12	**Mindfulness based cognitive therapy (MBCT)** The MBCT program a mixture of exercises/meditations such as Scanning of the entire body Meditation by sitting and walking *Mindful stretching *Exercises with a focus on cognitive behavioral techniques *Personal reflections of every day	8 weeks of face to face MBCT each lasting 2 h * Guided home practice with follow up sessions.	**Beck Depression Inventory**—measured depression **STAI—**measured anxiety **Disease activity**—CDAI measured Crohn's disease and SCCAI measured disease activity of ulcerative colitis. **IBDQ—**measured IBD specific HRQOL	*(+) outcomes with trait anxiety (*p* = 0.048) and depression (*p* = 0.027) in the MBCT intervention group after the intervention and at 6 months follow up.
Jedel et al. (2014)	RCT USA	Ulcerative colitis patients who were in remission.	**Initial** (1) 27/26 (2) 27/26 **2 months** (2) 27/26 **6 months** (2) 27/26 **12 months** (2) 27/26	**Mindful based stress reduction (MBSR)** The MBSR included *Meditation, *Scanning of the body *Yoga techniques *Personal reflections	8 weeks of MBSR intervention that spanned 2.5 h. * Homework assignment included 45 min/day of MBSR, for 6 days in a week with the help of a CD disc.	**Beck Depression Inventory**—measured depression **STAI -** measured anxiety **IBDQ—**measured IBD specific HRQOL **Inflammatory Biomarkers**– fecal calprotectin, cytokines, CRP	*Anxiety and depression were not associated with MBSR. *Post-hoc analysis revealed positive effect of MBSR in the subset of UC adults with increased stress (p <0.001).
Gerbarg et al. (2015)	RCT USA	IBD patients	**Initial** (1) 29/27 (2) 15/12 **6 weeks** (2) 15/12 **26 weeks** (2) 14/11	**BBMW** The participants were taught *4 breathing techniques (core breath technique, resistance breathing, breath moving and ‘Ha' breath) *Breathing synchronized with Qigong movements *Open focus meditation.	The BBMW was offered for 1.5 h for 6 weeks, then very month until week 26. *20-min breathing practice at home followed by a 3-min supine rest and were informed to keep a daily practice log. A CD provided.	**Beck Anxiety Inventory—**measured anxiety **Beck Depression Inventory**—measured depression **IBDQ—**measured IBD specific HRQOL **Inflammatory Biomarkers**– fecal calprotectin, and CRP	*(+) outcomes with anxiety at 6 weeks (*p* = 0.02) and at 26 weeks (*p* = 0.03). *(+) outcomes with depression at 26 weeks (*p* = 0.01).
Mizrahi et al. (2012)	RCT Israel	IBD patients	(1) 56/39 (2) 18/21	**Guided imagery with relaxation** The intervention consisted of *Different relaxation Guided imagery *Discussion of forms to monitor relaxation Brief review of struggles faced by patients to reach relaxation	Three individual relaxation training for 50 min ×5 weeks. *Home practice at least once a day for 5 weeks. A relaxation and guided imagery audio disc was provided.	**STAI—** measured anxiety **VAS**—measured depression **IBDQ—**measured IBD specific HRQOL	*(+) outcomes with anxiety (*p* <0.01). *No significant improvement noted with depression.
Vogelaar et al. (2014)	RCT Netherlands	IBD patients in remission	I**nitial** (1) 49/49 (2) 48/49 **At 3 months** (2) 48/49 **At 6 months** (2) 48/49	**SFT** SFT is a short version of psychotherapy focused on the present coping skills, instead of addressing the problems. The SFT was modified to addresses fatigue for this study.	Six group sessions (each lasted for 1.5 h) for 3 months' followed by a booster session at 6 months. A family member, partner or a close relative participated in the 5th session. *None	**HADS**—measured anxiety and depression **SF-36—**measured HRQOL **IBDQ—**measured IBD specific HRQOL **Inflammatory Biomarkers**– fecal calprotectin, and CRP	*(+) outcomes with depression (*p* = 0.03) in the SFT group at 3 months. The change in depression scores did not sustain after 3 months. * No difference in anxiety noted between the SFT and control group.
Cramer et al. (2017)	RCT Germany	Ulcerative colitis patients who were in remission	I**nitial** (1) 39/38 (2) 39/38 **At 12 weeks** (2) 27/34 **At 24 weeks** (2) 27/30	**Yoga** *Three trained yoga instructors taught Hatha yoga techniques. Each session has Exercises to loosen the body *Pre-defined yoga posture *Yogic breathing techniques (alternate nostril breathing) *Voiced breathing technique with a medication focus *Positive inhalation followed by forceful exhalation *Yogic meditation techniques (mantra meditation and Yoga nidra)	Yoga was administered 90 min per week /12 weeks *A manual was provided to each patient and they were instructed to practice at home on a daily basis. A daily log was provided to enter home practice at home.	**HADS**—measured anxiety and depression **SF-36—**measured HRQOL **Inflammatory Biomarkers**– fecal calprotectin, and CRP	*(+) outcomes for anxiety (*p* = 0.001) and for depression (*p* = 0.03) at 12 weeks for yoga group. *(+) outcomes for anxiety (*p*= 0.003) and depression (*p* = 0.007) at 24 months in yoga group.

### Meta-Analysis

The meta-analyses were computed using Comprehensive Meta-Analysis (CMA) software version 3 (Comprehensive Meta-Analysis, 2019) with the help of a University statistician. The authors used the CMA software to compute the efficacy of non-pharmacological interventions, to mitigate anxiety and depression in those with IBD, as well as to examine the influence of the interventions on QOL. The effect of non-pharmacological interventions on disease specific QOL (assessed using inflammatory bowel disease questionnaire [IBDQ]), and both mental and physical health QOL (evaluated by the short form health questionnaire [SF-12 or SF-36]) were assessed. Meta-analysis conclusions were drawn from pooled standardized mean difference**s** (SMD) and 95% confidence intervals (CI), which were estimated using random-effects models. The standardized mean difference in anxiety and depression between the treatment and control groups was computed for each study. The extent of heterogeneity between the selected studies was evaluated based on the *I*^2^ value. The *I*^2^ is a value that can range from 0 to 100%, where 0 indicates no heterogeneity and larger values denote marked heterogeneity (Higgins et al., 2003). The authors did not evaluate publication bias through examining the funnel plot nor by conducting the Egger test due to the limited number of studies (*N* < 10) available for meta-analysis.

## Results

### Characteristics of Included Studies

All of the selected studies reported a control group. The design of these control groups varied among the studies. Four of the selected studies (Schwarz and Blanchard, 1991; Sibaja et al., 2007; Mizrahi et al., 2012; Schoultz et al., 2015) had waitlist control groups; and two of them (Jedel et al., 2014; Gerbarg et al., 2015) had attention control groups. Control group participants were taught about IBD and its management (Gerbarg et al., 2015) and the influence of stress on sleep, psychological and physical health was the subject for teaching by Jedel et al. (2014). Three of the studies (Vogelaar et al., 2014; Mikocka-Walus et al., 2015; McCombie et al., 2016) used standard care as the control group. Lastly, one study (Cramer et al., 2017) used a combination of attention control (self-care advice with a focus on disease process, lifestyle changes and alternative treatments) and waitlist control groups.

Twelve articles were excluded. Five were not RCTs (Bregenzer et al., 2005; Evertsz et al., 2012; Jedel et al., 2013; Neilson et al., 2016; Jordan et al., 2019). Two were study protocols (Schoultz et al., 2013; van den Brink et al., 2016). Three were reviews (McCombie et al., 2013; Fiest et al., 2016; Taft et al., 2017), along with one qualitative study (Schoultz et al., 2016) and one study which included participants <18 years of age (Jantschek et al., 1998).

All of the selected studies reported exclusion or inclusion criteria and included both men and women; only one study was double-blinded (Jedel et al., 2014). Three of the articles (Jedel et al., 2014; Vogelaar et al., 2014; Mikocka-Walus et al., 2015; McCombie et al., 2016) demonstrated adequate sample size by reporting the power analyses. Only one study (Jedel et al., 2014) informed the details of blinding procedures. Objectives, outcomes, details of the intervention, and statistical outcomes were clearly addressed in all studies. Sample sizes ranged from 11 to 51 in experimental groups and 12–66 in control groups. The selected studies were from Spain, Australia, Netherlands, United Kingdom (UK), New Zealand, Israel, United States (US), and Germany.

Hospital Anxiety and Depression scale (HADS) was employed to measure anxiety and/or depression in five of the studies (Vogelaar et al., 2014; Mikocka-Walus et al., 2015 Sibaja et al., 2007; McCombie et al., 2016; Cramer et al., 2017). Other studies (Schwarz and Blanchard, 1991; Mizrahi et al., 2012; Schoultz et al., 2015) utilized the State Trait Anxiety Inventory (STAI) to evaluate anxiety, and some studies (Schwarz and Blanchard, 1991; Sibaja et al., 2007; Jedel et al., 2014; Gerbarg et al., 2015; Schoultz et al., 2015) used the Beck depression inventory to measure depression.

The majority of the RCTs examined the influence of non-pharmacological interventions on other health related outcomes. Seven studies (Mizrahi et al., 2012; Jedel et al., 2014; Vogelaar et al., 2014; Gerbarg et al., 2015; Schoultz et al., 2015; McCombie et al., 2016; Cramer et al., 2017) examined QOL using the inflammatory bowel disease questionnaire (IBDQ). QOL was also measured utilizing the short form 36 health status questionnaire (SF-36) (Vogelaar et al., 2014; Mikocka-Walus et al., 2015; Cramer et al., 2017) and the short form 12 (SF−12) health status questionnaire (McCombie et al., 2016). Biomarkers of inflammation, such as C-reactive protein (CRP), cytokines, and fecal calprotectin were also assessed in three of the selected studies (Vogelaar et al., 2014; Gerbarg et al., 2015; Cramer et al., 2017).

### Categories of Non-pharmacological Interventions Addressed in Studies

The selected ten articles addressed various non-pharmacological interventions to mitigate anxiety and depression among those with IBD (Table 2). The interventions included components of mindfulness-based therapy; cognitive behavioral therapy (CBT); breathing, movement, and meditation; guided imagery with relaxation; solution-focused therapy; education; a multicomponent regimen with muscle relaxation, biofeedback, coping skills, and education; and yoga.

**Table 2:** Analyses of interventions.

**Study name**	**TIME**	**Breathing**	**Qigong movement**	**Meditation**	**Home**	**Experiment/control group**	**Yoga**	**Relaxation**	**Guided imagery**	**Mindful stretching**	**Cognitive Behavior**	**Discussion reflection**	**Body scan**	**Education**	**SFT**
Gerbarg et al. (2015)	9 h ×2 Days (6 h day 1 and 3 days 2)	X	X	X	X (CD)	1.4-Exp / 11-Control									
Cramer et al. (2017)	90 min over 12 weeks	X (10 minu)		X (15)	X (Manual)	39-Exp / 38-Control	X						X (Yoga Nidra)		
Mizrahi et al. (2012)	3–50 min Sessions every 2 weeks (total 6 weeks)	X			X (CD)	18-Exp / 21-Control		X (Muscle)	X						
Schoultz et al. (2015)	8 Weekly F2F- 2 h each			X	X (CD/Manual)	12- / 12-				X	X	X	X		X
Jedel et al. (2014)	FLAIR 8 Weeks 1X/week for 2–2.5 h			X	X (CD)	No flare 12/ 10 control and flare 4/ 3 control		X (Posture)			X (Awareness mindfulness)		X		
Sibaja et al. (2007)	10 Weeks 2 hours/ weeks	X				33-Exp / 14-Control		X (Muscle)			X (Problem Solving Att./Distraction)			X (On Relaxation IBD Coping Communication. Assertiveness.)	
McCombie et al. (2016)	8 Sessions-−62 resources self-paced over 8 weeks (computer access)					Completed 50% 29-Exp / 66-Control		X			X (Distraction Thoughts /Behavior)			X (Communication Coping)	
Mikocka-Walus et al. (2015) Pilot	10 weeks F2F and Online					6 Months 55-Exp / 65-Control (42-Exp / 64-Control)		X			X (Att. Distraction)			X (IBD / CPT Assertive Coping Strategies, Comm/Relationships, relapse prevention)	
Mikocka-Walus et al. (2017) 24 Months	10 week Group 2 hours/Week					24 Months 30-Exp / 45-Control									
Vogelaar et al. (2014)	6 group sessions- 1.5 hrs X 3 months; a booster session at 6 months					6 months- 48 exp/49 control									Focused on the present coping abilities
Schwarz and Blanchard (1991)	F 2 F two session ×4 weeks and one session ×4 weeks							X (Muscle)			X			X (IBD)	

Intervention length and dose differed significantly between studies, ranging from 2 days to 12 weeks. Six of the studies had a home component to the intervention. Three included a computer disc (Mizrahi et al., 2012; Jedel et al., 2014; Gerbarg et al., 2015), one had a manual (Cramer et al., 2017), and two had both disc and manual (Sibaja et al., 2007; Schoultz et al., 2015). Additionally, researchers of one study sent emails or text messages to participants in between sessions as a reminder to engage in the suggested interventions (McCombie et al., 2016).

Each study intervention was examined for components. All of the reviewed studies had multiple components to each intervention. The most common component of the interventions found in five of the studies was relaxation, including muscle relaxation and guided imagery (Schwarz and Blanchard, 1991; Sibaja et al., 2007; Mizrahi et al., 2012; Jedel et al., 2014; Mikocka-Walus et al., 2015; McCombie et al., 2016). Different relaxation techniques were noted across the studies. Meditation was incorporated as part of the interventions in four studies (Jedel et al., 2014; Gerbarg et al., 2015; Schoultz et al., 2015; Cramer et al., 2017), followed by three studies employing breathing techniques (Mizrahi et al., 2012; Gerbarg et al., 2015; Cramer et al., 2017), as well as the use of mental body scanning in three (Jedel et al., 2014; Schoultz et al., 2015; Cramer et al., 2017) studies. Meditation was recorded as part of yoga (Cramer et al., 2017), mindful movements including Qigong movement (Gerbarg et al., 2015), sitting meditation (Jedel et al., 2014), and mindful stretching (Schoultz et al., 2015). Cramer et al. (2017) concentrated on yoga nidra as a technique to center their attention on various body parts. Different yoga postures were incorporated into the interventions by both Cramer et al. (2017) and Jedel et al. (2014).

Cognitive behavior interventions were noted in four studies (Schwarz and Blanchard, 1991; Sibaja et al., 2007; Mikocka-Walus et al., 2015; Schoultz et al., 2015; McCombie et al., 2016). The techniques included awareness and mindfulness training, problem-solving, attention distraction (Sibaja et al., 2007; Mikocka-Walus et al., 2015; McCombie et al., 2016), and cognitive restructuring (Sibaja et al., 2007; Mikocka-Walus et al., 2015). Education was a component of four studies (Schwarz and Blanchard, 1991; Sibaja et al., 2007; Vogelaar et al., 2014; Mikocka-Walus et al., 2015). Education included information on coping with IBD, communication and assertiveness, relaxation, and relapse prevention. All of these studies addressed communication, while three addressed coping (Schwarz and Blanchard, 1991; Sibaja et al., 2007; Vogelaar et al., 2014; McCombie et al., 2016). Vogelaar et al. (2014) implemented solution-focused therapy (SFT) as an intervention to mitigate depression and anxiety in adults with IBD which focused on the present coping abilities of the patient.

### Effectiveness of Non-pharmacological Interventions

#### Meta-Analysis Results

Meta-analysis was conducted to generate pooled SMD and confidence intervals (CI) for intervention effects on anxiety, depression, disease specific QOL which was measured by IBDQ, mental, and physical QOL which were measured by SF-12/SF-36. In fixed effects models, the *I*^2^ values ranged from 0.0 to 0.74 indicating low to moderate heterogeneity. Despite the fact that some values were low, we report random effects models, as between-study heterogeneity is to be expected given the diverse populations and interventions.

Seven of the 10 studies presented enough quantitative data to compute a meta-analysis to evaluate the influence of non-pharmacological interventions on anxiety and depression (Mizrahi et al., 2012; Jedel et al., 2014; Gerbarg et al., 2015; Mikocka-Walus et al., 2015; Schoultz et al., 2015; McCombie et al., 2016; Cramer et al., 2017). We estimated the pooled standardized mean differences (SMD) between the non-pharmacological intervention and control groups for both anxiety and depression.

**Figure 3:**
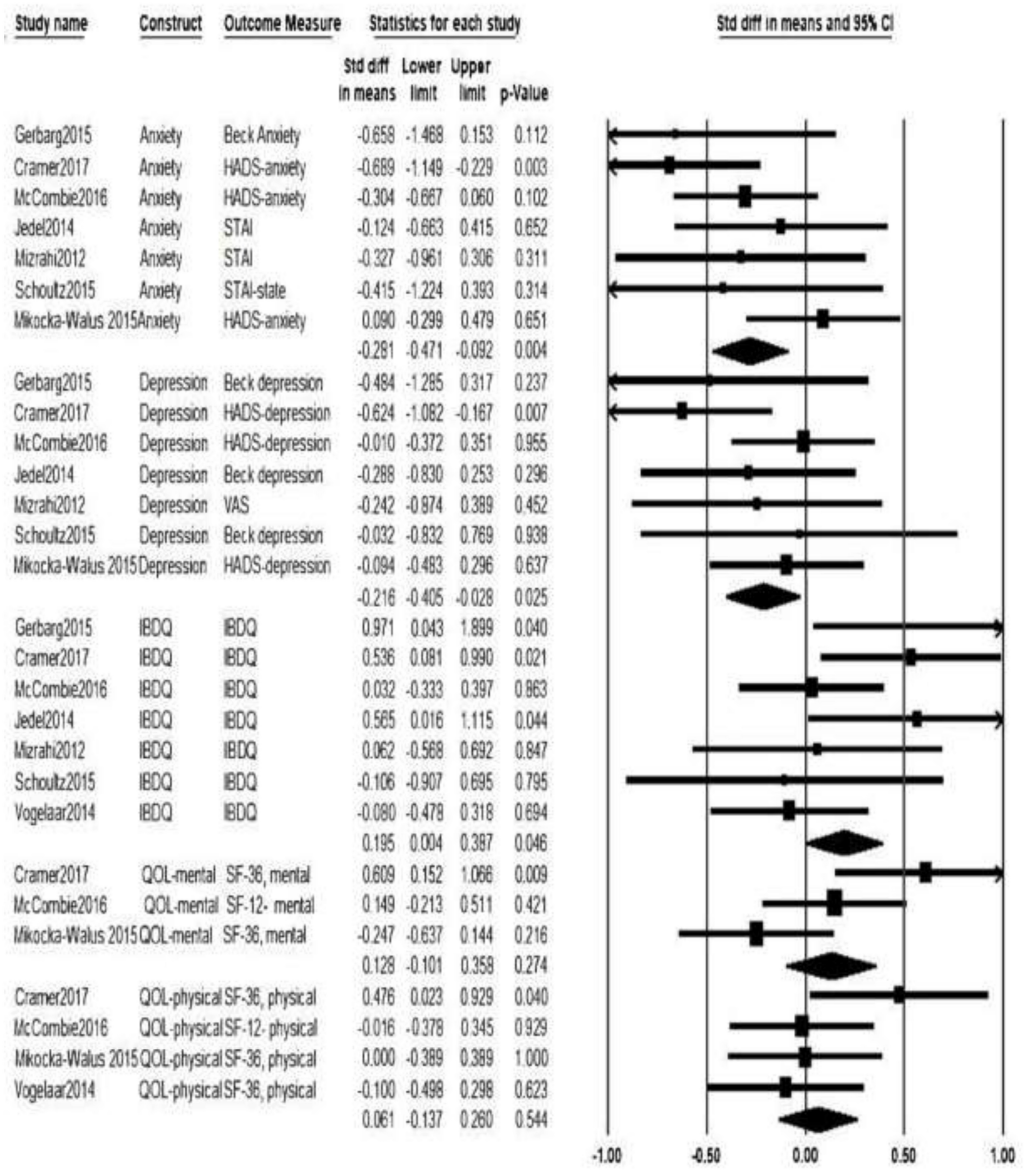
Forest plot with meta–analysis results. HADS, Hospital Anxiety and Depression Scale; STAI, State Trait Anxiety Inventory; VAS- Visual Analog Scale; IBDQ, Inflammatory Bowel Disease Questionnaire; QOL, Quality of Life; SF-36, The Short Form 36 health status questionnaire; SF-12, The Short Form 12 health status questionnaire.

The forest plot (Figure 3) shows the individual study estimated effects on each outcome, along with the pooled SMD from the random effects models. The SMDs represent the standardized mean difference in the change over time (pre to post-intervention) for the intervention vs. control group. When multiple time points were available, we used the one most immediately following the end of the intervention, which ranged from 5 weeks to 12 months from baseline.

For the effect of non-pharmacological interventions on anxiety, the pooled standardized mean differences (SMD) was −0.28 (95% CI [−0.47, −0.09], *p* = 0.004). This suggests that, on average, non-pharmacological interventions from seven RCTs significantly reduced anxiety among adults with IBD by about ¼ of a standard deviation. The pooled SMD was −0.22 (95% CI [−0.41, −0.03], *p* = 0.025) for the effect of non-pharmacological interventions on depression, indicating that non-pharmacological interventions significantly helped to reduce the depression among adults with IBD. Refer to Figure 2 for forest plot.

We fit random effects models to examine the efficacy of non-pharmacological interventions on QOL. Seven studies (Mizrahi et al., 2012; Jedel et al., 2014; Vogelaar et al., 2014; Gerbarg et al., 2015; Schoultz et al., 2015; McCombie et al., 2016; Cramer et al., 2017) measured disease specific QOL using IBDQ. Pooled SMD revealed a significantly higher IBDQ in the intervention group, suggestive of improved disease specific QOL in the intervention groups, with a standardized mean differences value of 0.20 (95% CI [0.004, 0.39], *p* = 0.046). Three of the selected studies (Mikocka-Walus et al., 2015; McCombie et al., 2016; Cramer et al., 2017) examined the impact of non-pharmacological intervention on the mental health component of QOL. The pooled effect on mental health QOL (using SF-12 or SF-36) was 0.13 (95% CI [−0.10, 0.36], *p* = 0.27). Four studies measured non-pharmacological intervention's effect on the physical health component of QOL (Vogelaar et al., 2014; Mikocka-Walus et al., 2015; McCombie et al., 2016; Cramer et al., 2017). For physical health QOL (assessed by the SF-12 or SF-36), the pooled SMD was 0.06 (95% CI [−0.14, 0.26], *p* = 0.54). Although the results of pooled SMD estimates for mental and physical QOL are positive (favoring the treatment group), neither is statistically significant.

### Synthesis of Two Studies

Findings of three of the selected studies (Schwarz and Blanchard, 1991; Sibaja et al., 2007; Vogelaar et al., 2014) were synthesized narratively due to insufficient quantitative data for meta-analysis for the outcomes of anxiety and depression. The interventions implemented were multicomponent regimens (Schwarz and Blanchard, 1991), CBT (Sibaja et al., 2007) and solution-focused therapy (Vogelaar et al., 2014). Both CBT and solution-focused therapy significantly reduced depression (Sibaja et al., 2007, *p* < 0.001; Vogelaar et al., 2014, *p* = 0.03). However, only CBT was effective in reducing anxiety which was persistent at 12 months after the intervention (Sibaja et al., 2007, *p* < 0.001). No statistically significant change in anxiety or depression scores was noted among the participants of the multicomponent regimen (Schwarz and Blanchard, 1991).

## Discussion

The goal of this systematic review and these meta-analyses were to examine RCTs of non-pharmacological interventions and their effectiveness for managing anxiety and depression in adults with IBD. This systematic review analyzed the evidence from a variety of interventions (CBT, yoga, guided imagery with relaxation, SFT, mindful-based stress reduction, and breath–body- mind –workshop [BBMW]) to mitigate anxiety and depression among adults with IBD. Unlike the findings of a prior systematic review (Fiest et al., 2016), the evidence synthesized from this systematic review and meta-analysis supports different non-pharmacological interventions, as the pooled effect of these interventions were found to be beneficial in reducing depression and anxiety among adults with IBD.

Another important result was that the pooled effect from IBDQ data supported the finding that non-pharmacological interventions improved disease specific QOL of adults with IBD. Previous findings confirmed the close association between depression, anxiety, and QOL of those with IBD. Depression and anxiety scores significantly lowered the QOL scores of adults with IBD (Evertsz et al., 2012). In light of this findings, non-pharmacological interventions to manage depression and anxiety in those with IBD ultimately help to improve their QOL and should be promoted.

Because a subgroup analysis was not possible due to the limited number of studies, we conducted a qualitative synthesis of individual interventions to understand how interventions influenced depression and fatigue. Among the different interventions, CBT was the most commonly used intervention (Sibaja et al., 2007; Mikocka-Walus et al., 2015; McCombie et al., 2016) followed by mindful based interventions (Jedel et al., 2014; Schoultz et al., 2015). The remaining interventions varied among the studies. However, examining the components of interventions revealed the use of several common therapeutic modalities (Table 2) in each of the reviewed RCTs. For example, as displayed in Table 2, cognitive behavioral interventions, relaxation, meditation, breathing, body scan and education were common therapeutic modalities as part of different interventions in many studies. This finding supports incorporating various therapeutic components to manage depression and anxiety among adults with IBD, as suggested by Knowles et al. (2013). Of the seven studies that evaluated QOL, three of them (Jedel et al., 2014; Gerbarg et al., 2015; Cramer et al., 2017) demonstrated statistically significant differences after the intervention. The interventions included in these studies were yoga (Cramer et al., 2017), breath–body- mind–workshop (BBMW; Gerbarg et al., 2015) and mindful based interventions (Jedel et al., 2014).

Out of 10, six of the reviewed studies included all adults with IBD and did not differentiate if the IBD was in remission or active state. Significant improvement was noted on both anxiety and depression in three of the studies (Sibaja et al., 2007; Gerbarg et al., 2015; Schoultz et al., 2015) and improvement was noted on anxiety in only one study (Mizrahi et al., 2012). Two studies did not report changes in depression and/or anxiety. Two studies were conducted among adults with IBD in remission and significant improvement was noted on both anxiety and depression in one study (Mikocka-Walus et al., 2015), and only on depression in the second study (Vogelaar et al., 2014). Two studies included participants with ulcerative colitis (a subtype of IBD) in remission, and the findings of only one study (Cramer et al., 2017) noted an improvement in anxiety and depression.

Not only did the interventions differ, but the dose and strength of the interventions differed. Although the evidence is supportive of non-pharmacological interventions to manage anxiety and depression in adults with different types of IBD, further research is recommended with more studies to identify the distinct evidence on adults with different types of IBD.

All the studies included in this synthesis had longitudinal assessments of outcomes to determine if effects were sustained. Sustained improvement on anxiety and depression outcomes was present only in the results of the study by Sibaja et al. (2007); however, the control group of this study was included only for initial assessment, with no comparison between treatment and control groups at three, six, and 12 months. Further research is warranted with the inclusion of treatment and control groups in follow-ups to determine the persistent effects of non-pharmacological interventions.

All the studies except one (Gerbarg et al., 2015) included some form of initial psychological screening as inclusion criteria before initiating the non-pharmacological interventions in their RCTs, forming a homogeneous group of study participants among the eight studies regarding their mental health. However, Jedel et al. (2014) acknowledged that participants' self-reporting of sound baseline mental health status might have caused a ceiling effect on the results of the study and recommended the consideration of baseline mental status as a sampling criterion.

The setting varied across the studies from Spain, Australia, New Zealand, UK, US, Israel, Netherlands, and Germany. Therefore, it is important to consider the acceptability and feasibility of various non-pharmacological interventions across diverse healthcare settings and cultures. Overall, the review presented a variety of non-pharmacological modalities to mitigate anxiety and depression and noted the positive effects of most of these therapeutic modalities on the anxiety and depression outcomes of those with IBD.

Few studies documented the type of reinforcement or reminder about the interventions to enhance compliance or to improve the effects of interventions. This synthesis found multiple studies using a home component as part of the intervention. However, neither the dosage, adherence, nor timing of intervention were clearly detailed. The majority of the researchers used the same methodology as Vogelaar et al. (2014) and included an extended treatment period with a follow- up to maintain a persistent effect of non-pharmacological interventions in those with IBD.

Some limitations were observed among the studies selected for review. We noticed a heterogeneous control groups among the studies that included in meta-analysis which varied from waitlist control groups, attention control groups, and standard control groups. Therefore, the results of the meta-analysis need to be interpreted with caution due to the different control groups of the reviewed studies. Many studies did not evaluate anxiety or depression as a primary outcome. Most of them assessed anxiety or depression along with other variables such as quality of life, physical symptoms, inflammatory biomarkers, and stress. Due to this weakness, further research is recommended to evaluate the distinct contribution of non-pharmacological interventions on anxiety and depression in adults with IBD.

Additionally, the disease activity of samples varied across the studies. They included IBD in general, IBD in remission, ulcerative colitis with remission, and those with active IBD. Moreover, no consistency was observed among the delivered non-pharmacological interventions in these different samples with IBD disease activity. Consequently, it was difficult to conclude the suitability of a particular intervention for certain disease activity of IBD. More research targeting various disease activities is required to understand the effectiveness of these interventions on the various disease activities of IBD.

Failure to retain participants after six and 12 months were an observed problem among many studies (Mikocka-Walus et al., 2015; Schoultz et al., 2015; McCombie et al., 2016; Cramer et al., 2017). This might have affected the results with long-term effects of non-pharmacological interventions in these studies; future research should be designed to improve participant retention with longitudinal assessment of anxiety and depression.

## Conclusion and Implications for Practice

Analysis of the reviewed RCTs indicates that non-pharmacological interventions can be an option for the management of anxiety and depression among adults with IBD. Non-pharmacological interventions can be considered as concurrent therapy with pharmacological treatment to manage depression and anxiety in adults with IBD. Thus, all healthcare providers in the acute or outpatient care settings should facilitate a discussion with adults with IBD about the availability of these interventions to manage their anxiety and depression. In conclusion, providers should screen adults with IBD based on the disease activity of IBD and refer them to the appropriate non-pharmacological intervention of their choice, as anxiety and depression have been related to the disease recurrence in the IBD population (Mikocka-Walus et al., 2016).

## Data Availability Statement

The datasets generated for this study are available on request to the corresponding author.

## Author Contributions

SD contributed to the conception, design, systematic search, data extraction, quality appraisal, and analysis. LB and PC contributed to the design, data extraction, quality appraisal, analysis, and critical revision of the draft. JC contributed to meta-analysis, interpretation of meta-analysis results, and critical revision of the draft. Finally, all authors have reviewed and approved the final paper.

## Conflict of Interest

The authors declare that the research was conducted in the absence of any commercial or financial relationships that could be construed as a potential conflict of interest.
