# Supplementary material for: Non-pharmacological Interventions for Anxiety and Depression in Adults With Inflammatory Bowel Disease: A Systematic Review and Meta-Analysis
Source: Front Psychol. 2020 Nov 5;11:538741. doi: 10.3389/fpsyg.2020.538741 (PMC7676293; doi:10.3389/fpsyg.2020.538741)
Supplement: Supplementary file 1 [file Table_1.docx]

**Supplementary Table 1**

*Search Strategy*

Database: PubMed (MEDLINE)

| Set # |  | Results |
| --- | --- | --- |
| 1 | "Inflammatory Bowel Diseases"[Mesh] OR “inflammatory bowel disease”[tiab] OR “inflammatory bowel diseases”[tiab] OR “Ulcerative Colitis”[tiab] OR “Crohn Disease”[tiab] OR “Crohn's Disease”[tiab] OR “Crohns Disease”[tiab] OR “Crohn's Enteritis”[tiab] | 110437 |
| 2 | "Anxiety"[Mesh] OR "Anxiety Disorders"[Mesh] OR "Depression"[Mesh] OR "Depressive Disorder"[Mesh] OR anxiety[tiab] OR anxieties[tiab] OR anxious[tiab] OR depressive[tiab] OR depression[tiab] OR depressions[tiab] OR depressed[tiab] | 628665 |
| 3 | #1 AND #2 | 1679 |
| 4 | #3 AND English[lang] | 1522 |
| 5 | #4 AND (randomized controlled trial[pt] OR controlled clinical trial[pt] OR randomized[tiab] OR placebo[tiab] OR drug therapy[sh] OR randomly[tiab] OR trial[tiab] OR groups[tiab]) NOT (animals [mh] NOT humans [mh]) | 535 |

Database: CINAHL

| Set # |  | Results |
| --- | --- | --- |
| 1 | MH "Inflammatory Bowel Diseases+" OR TI (“inflammatory bowel disease” OR “inflammatory bowel diseases” OR “Ulcerative Colitis” OR “Crohn Disease” OR “Crohn's Disease” OR “Crohns Disease” OR “Crohn's Enteritis”) OR AB (“inflammatory bowel disease” OR “inflammatory bowel diseases” OR “Ulcerative Colitis” OR “Crohn Disease” OR “Crohn's Disease” OR “Crohns Disease” OR “Crohn's Enteritis”) | 19648 |
| 2 | MH "Anxiety Disorders+" OR MH "Anxiety+" OR MH "Depression+" OR TI (anxiety OR anxieties OR anxious OR depressive OR depression OR depressions OR depressed) OR AB (anxiety OR anxieties OR anxious OR depressive OR depression OR depressions OR depressed) | 265994 |
| 3 | #1 AND #2 | 556 |
| 4 | #3 AND English | 538 |
| 5 | #4 AND (MH "Randomized Controlled Trials" OR MW “DT” OR TI (randomized OR placebo OR randomly OR trial OR groups) OR AB (randomized OR placebo OR randomly OR trial OR groups) | 194 |

Database: PsycINFO

| Set # |  | Results |
| --- | --- | --- |
| 1 | DE "Ulcerative Colitis" OR TI (“inflammatory bowel disease” OR “inflammatory bowel diseases” OR “Ulcerative Colitis” OR “Crohn Disease” OR “Crohn's Disease” OR “Crohns Disease” OR “Crohn's Enteritis”) OR AB (“inflammatory bowel disease” OR “inflammatory bowel diseases” OR “Ulcerative Colitis” OR “Crohn Disease” OR “Crohn's Disease” OR “Crohns Disease” OR “Crohn's Enteritis”) | 1595 |
| 2 | DE "Anxiety" OR DE "Anxiety Sensitivity" OR DE "Computer Anxiety" OR DE "Health Anxiety" OR DE "Mathematics Anxiety" OR DE "Performance Anxiety" OR DE "Social Anxiety" OR DE "Speech Anxiety" OR DE "Test Anxiety" OR DE "Anxiety Disorders" OR DE "Castration Anxiety" OR DE "Death Anxiety" OR DE "Generalized Anxiety Disorder" OR DE "Obsessive Compulsive Disorder" OR DE "Panic Attack" OR DE "Panic Disorder" OR DE "Phobias" OR DE "Separation Anxiety Disorder" OR DE "Trichotillomania" OR DE "Major Depression" OR DE "Anaclitic Depression" OR DE "Dysthymic Disorder" OR DE "Endogenous Depression" OR DE "Late Life Depression" OR DE "Postpartum Depression" OR DE "Reactive Depression" OR DE "Recurrent Depression" OR DE "Treatment Resistant Depression" OR DE "Depression (Emotion)" OR TI (anxiety OR anxieties OR anxious OR depressive OR depression OR depressions OR depressed) OR AB (anxiety OR anxieties OR anxious OR depressive OR depression OR depressions OR depressed) | 449190 |
| 3 | #1 AND #2 | 378 |
| 4 | #3 AND English | 322 |
| 5 | #4 AND DE "Treatment Effectiveness Evaluation" OR DE "Common Factors" OR DE "Treatment Outcomes" OR DE "Psychotherapeutic Outcomes" OR DE "Side Effects (Treatment)" OR DE "Treatment Compliance" OR DE "Treatment Duration" OR DE "Treatment Refusal" OR DE "Treatment Termination" OR DE "Treatment Withholding" OR DE "Placebo" OR DE "Randomized Controlled Trials" OR DE "Randomized Clinical Trials" OR DE "Followup Studies" OR placebo* OR random* OR "comparative stud*" OR clinical NEAR/3 trial* OR research NEAR/3 design OR evaluat* NEAR/3 stud* OR prospectiv* NEAR/3 stud* OR (singl* OR doubl* OR trebl* OR tripl*) NEAR/3 (blind* OR mask*) | 39 |

Database: Embase

| Set # |  | Results |
| --- | --- | --- |
| 1 | 'inflammatory bowel disease'/exp OR ‘inflammatory bowel disease’:ti,ab OR ‘inflammatory bowel diseases’:ti,ab OR ‘Ulcerative Colitis’:ti,ab OR ‘Crohn Disease’:ti,ab OR ‘Crohns Disease’:ti,ab | 180163 |
| 2 | 'anxiety'/exp OR 'anxiety disorder'/exp OR 'depression'/exp OR anxiety:ti,ab OR anxieties:ti,ab OR anxious:ti,ab OR depressive:ti,ab OR depression:ti,ab OR depressions:ti,ab OR depressed:ti,ab | 1,027, 450 |
| 3 | #1 AND #2 | 4676 |
| 4 | #3 AND [english]/lim | 4438 |
| 5 | #4 AND 'crossover procedure':de OR 'double-blind procedure':de OR 'randomized controlled trial':de OR 'single-blind procedure':de OR (random* OR factorial* OR crossover* OR cross NEXT/1 over* OR placebo* OR doubl* NEAR/1 blind* OR singl* NEAR/1 blind* OR assign* OR allocat* OR volunteer*):de,ab,ti | 610 |
| 6 | #6 AND [embase]/lim NOT ([embase]/lim AND [medline]/lim) | 270 |
